# 

**Published:** 1860-11

**Authors:** 


### CONTENTS.

ORIGINAL ESSAYS AND COMMUNICATIONS.

The Province of the Dentist. By J. Taft, ..... 249
Purple of Cassius. By Prof. William Calvert, D. D. S. ..... 254
Extortion ..... 256
The Anatomy, Physiology, Pathology, and Remedial Treatment of the Fifth Pair of Nerves. By J. H. M'Quillen, D. D. S. ..... 259
Hypertrophy ..... 267
Thoughts on Dental Caries. By Geo. Watt ..... 270
Biographical Sketch of Dr. Alvin Blakesley, of Utica, N. Y. ..... 275

PROCEEDINGS OF SOCIETIES.

Proceedings of the Local Dental Association ..... 278
Selections ..... 281
Correspondence ..... 306
Editorial ..... 308

Communications must reach the Editor as early as the 15th, to insure insertion in the next issue.

